# Repeated Working Memory Training Improves Task Performance and Neural Efficiency in Multiple Sclerosis Patients and Healthy Controls

**DOI:** 10.1155/2019/2657902

**Published:** 2019-04-16

**Authors:** Naiara Aguirre, Álvaro Javier Cruz-Gómez, Anna Miró-Padilla, Elisenda Bueichekú, Ricardo Broseta Torres, César Ávila, Carla Sanchis-Segura, Cristina Forn

**Affiliations:** ^1^Universitat Jaume I. Departament de Psicología Bàsica, Clínica i Psicobiología, Castelló de la Plana 12006, Spain; ^2^ERESA, Grupo Médico., Valencia 46015, Spain

## Abstract

**Background/Objective:**

To explore the effectiveness of a specific working memory (WM) training program in MS patients and healthy controls (HC).

**Method:**

29 MS patients and 29 matched HC were enrolled in the study. MS and HC were randomly split into two groups: nontraining groups (15HC/14 MS) and training groups (14 HC/15 MS). Training groups underwent adaptive n-back training (60 min/day; 4 days). Functional magnetic resonance imaging (fMRI) was used to monitor brain activity during n-back performance (conditions: 0-back, 2-back, and 3-back) at 3 time points: (1) baseline, (2) post-training (+7days), and (3) follow-up (+35days).

**Results:**

In post-training and follow-up fMRI sessions, trained groups (HC and MS patients) exhibited significant reaction time (RT) reductions and increases in Correct Responses (CRs) during 2-back and 3-back performance. This improvement of task performance was accompanied by a decrease in brain activation in the WM frontoparietal network. The two effects were significantly correlated.

**Conclusions:**

After WM training, both cognitively preserved MS patients and HC participants showed task performance improvement made possible by neuroplastic processes that enhanced neural efficiency.

## 1. Introduction

Cognitive impairment is present in 40-65% of Multiple Sclerosis (MS) patients [1]. Although the neuropsychological profile varies among patients, there is evidence that the functions mainly affected in this clinical population, even in the early stages of the disease, are information processing speed, attention, and working memory (WM) [2, 3]. A decline in cognitive functions has a negative impact on patients' daily lives and quality of life [3]. Consequently, numerous studies have been carried out in recent years to explore the potential effects of cognitive interventions focused on improving cognition in MS patients.

In this regard, several studies using functional Magnetic Resonance Imaging (fMRI) have provided positive evidence about the presence of neural plasticity in MS patients that helps to conceal the disease's clinical expression, at least in some phases of the disease [4–6]. However, the counteracting effects of spontaneous neuroplasticity are limited and transitory, and they are ultimately overcome by the disease's progression [7].

As initial evidence of this concept, Sastre Garriga et al. [8] showed that neuropsychological training resulted in general cognitive improvement in MS patients, with an increase in activity in cerebellar areas. After this initial study, other researchers showed the efficacy of neuropsychological interventions in improving memory [9–11], attention, processing information speed, and executive functions [12–17] in MS patients. In general, the results of these studies revealed greater cerebral activation or higher functional connectivity in MS patients after completing the training program, which in some cases was positively correlated with improvements in cognitive performance. However, as a recent review pointed out [18], the results of these studies are inconclusive, perhaps due to the heterogeneity of the selected participants, the diversity and lack of specificity of the rehabilitation approaches used, and other methodological weaknesses (e.g., the selection of outcome measures).

Taking these limitations into account, we designed a study to test the effectiveness of a WM training program in a group of MS patients. We focused our cognitive training on WM functions because it has been suggested that cognitive rehabilitation programs targeting specific cognitive domains could maximize their effectiveness; and improving functions that play central roles in the cognitive architecture (as in the case of WM and cognitive executive control processes) will maximize the applicability of the intervention's effect (i.e., generality) [18]. From the experimental tasks that could be used to assess and train WM functions, we chose the n-back because it has been found to improve WM [19, 20]. Taking into account that WM processes involve the maintenance and fast manipulation of information, training WM processes can also lead to an improvement in information processing speed. Finally, regarding participant selection, we recruited healthy controls (HC) and a very homogenous sample of relapsing-remitting (RR) MS patients without signs of cognitive deficits. This experimental design was selected in order to (1) ensure that there were no initial (baseline assessment) cerebral differences in MS patients secondary to their pathology; (2) allow between but also within-group comparisons that could corroborate possible WM improvement; and (3) assess whether the cerebral changes associated with WM training are similar or different in HC and MS patients.

Taking into account previous studies reporting that repeated task training produces performance enhancements and brain activity decreased in HC [20], the aim of the present study was to explore whether n-back training could improve task performance in MS patients and decrease cerebral activity in WM networks indicative of neural efficiency.

## 2. Material and Methods

### 2.1. Participants

Twenty-nine right-handed patients diagnosed with clinically definite RR MS according to McDonald's criteria [21] were selected for the study, and twenty-nine right-handed participants with no neurological or psychiatric dysfunction made up the control group (HC). Participants were randomly subdivided into four groups: 14 MS untrained group (MSu), 15 HC untrained group (HCu), 15 MS trained group (MSt), and 14 HC trained group (HCt). All participants received remuneration for completing the study. The Ethical Committee of Universitat Jaume I approved the research project. All participants gave informed written consent prior to participation.

**Figure 1:**
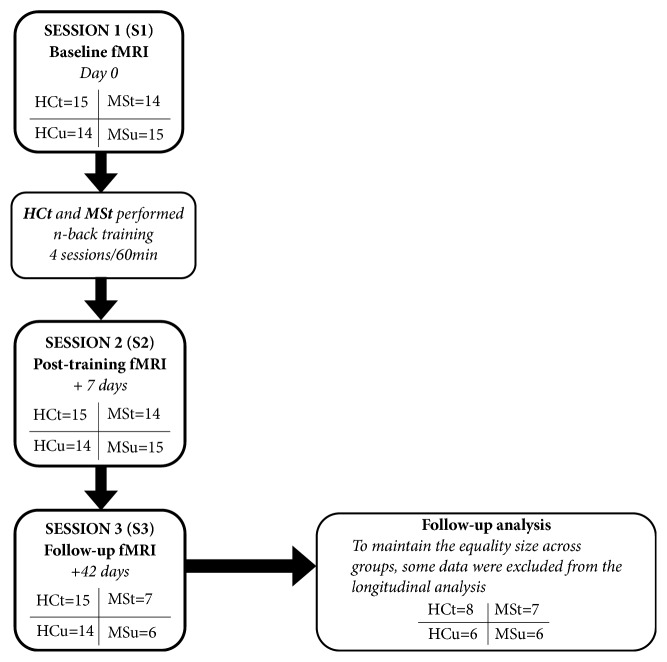
Flow diagram of the phases of the study.

All participants were assessed with (1) the Brief Repeatable Battery of Neuropsychological Tests (BRB-N), validated for the Spanish population [22]; the Matrix Reasoning Subtest of the Wechsler Adult Intelligence Scale (WAIS III) to assess the intelligence quotient (IQ); (2) the Fatigue Severity Scale (FSS); and (3) the Beck Depression Inventory (BDI). In addition, patients were neurologically assessed using the Expanded Disability Status Scale (EDSS). Moreover, assessment also included three functional Magnetic Resonance Imaging (fMRI) sessions: base-line session (S1), post-training session (S2; 7 days later), and follow-up session (S3; 42 days later). In S3, due to technical problems, the data of 7 MSt and 9 MSu participants were lost. Therefore, we randomly dropped data from 7 HCt and 8 HCu in order to compare similarly sized groups (8 HCu/6 HCt/7 MSu/6 MSt). See Figure 1 for a schematic description of the experimental design.

### 2.2. MRI Acquisition

MRI data were collected on a 1.5T scanner (Siemens Symphony, Erlangen, Germany) in the following order in the three sessions: (1) anatomical 3D MPRAGE volumes were acquired, using a T1-weighted gradient echo pulse sequence (TR=2200ms; TE=3ms; flip angle=15°; matrix=256x256x160; voxel=1x1x1mm) and for MS patients a FLAIR sequence (TR=6000ms; TE=354ms; flip angle=180°; matrix=196x256x160; voxel=1.05x1.05x1mm); (2) fMRI data during n-back were acquired with a gradient-echoT2*∗*-weighted echo-planar MR sequence covering the entire brain (TR=2500ms; TE=49ms; matrix=64×64x28; flip angle=90°; voxel=3.5×3.5×3.5; slice gap=4.41mm). A total of 260 volumes were recorded.

### 2.3. N-Back fMRI Task

The n-back adapted for fMRI has been described in previous studies [20, 23]. Briefly, the n-back task used in this fMRI study consisted of a block task with 3 conditions: 0-back as a baseline control task and 2 and 3-back as a WM task. Visual stimuli comprised 15 capital letters from the alphabet; there were 270 stimuli in the entire task, and 54 were targets. Any letter could be the target in 2 and 3-back, but the X was the target in 0-back. Thus, during 0-back, subjects were instructed to press the “yes” button when the X target letter was presented on the screen, and the “no” button when any other letters were presented. During the 2 and 3-back tasks, participants were instructed to press the “yes” button when the letter presented on the screen matched the one presented 2 or 3 items back and press “no” when they saw no target letters on the screen. Manual responses were given with the right hand, responding to targets with their thumb and to nontargets with their forefinger. The task was composed of 9 blocks pseudo randomly presented, three for each level. Each block lasted 60.7s and consisted of 200ms of a blank screen, followed by 30 (6 target) consecutive trials of single-letter stimuli presented for 500ms with a 1500ms inter stimulus interval, with 500ms of a blank screen at the end of each block. In addition, each block also included 800ms of a fixation cross and 2000ms of the instruction display indicating the task difficulty of the block. The total duration of the task was 11 min. Before fMRI acquisition, subjects received oral instructions about how to do the task, and they performed 5 min practice. For a more specific description of the task, see Miró-Padilla et al. [20].

Visual stimuli were presented electronically using E-Prime software (Psychology Software Tools, Pittsburgh, PA), professional version 2.0, installed in a Hewlett-Packard portable workstation (screen-resolution 800 x 600, refresh rate of 60 Hz). Participants watched the laptop screen through MRI-compatible goggles (VisuaStim, Resonance Technology, Inc., Northridge, CA, USA). Participants had to make “yes” or “no” motor responses during the task that were collected via MRI-compatible response-grips (NordicNeuroLab, Bergen, Norway). E-Prime's logfile saved the correct responses (CRs) and reaction times (RTs) to each stimulus for each participant.

### 2.4. N-Back Training Protocol

Two days after S1, the trained groups came to the university for 4 sessions (duration: 60 min/session) of n-back training. These sessions were conducted, following the procedure reported by Miró-Padilla et al. [20], by three experimenters blinded to group (HC/ MS) assignment. Trained groups performed four training sessions of 60 min each on four consecutive days. Training sessions were distributed in two phases. During the first phase, participants performed WM training, which consisted of an adaptive n-back paradigm adapted from Jaeggi et al. [24] for 50 minutes. In this phase, participants performed three runs, each composed of eight blocks that varied in WM load (1-back, 2-back, and 3-back). For motivational reasons [25], the training always started at the low level, that is, with a 1-back load, but the level of n-back of the subsequent block was based on the participant's performance on the previous block. Thus, if the participant had at least 90% CRs, the WM load increased one level (e.g., 90% performance on 2-back tasks increased to 3-back). If the CRs during the block were below 80%, in the subsequent block the WM load decreased one level (e.g., from 2-back to 1-back). In all other cases, the n-level remained constant [26]. As in the n-back fMRI task, participants were instructed to give manual responses only with their right hand, responding to targets with their thumb and to nontargets with their forefinger. Feedback was introduced after each response for a few seconds, as a colored circle at the corner of the screen: green meant a correct answer, a red circle represented an error, and blue meant missing responses. Moreover, at the end of each block, subjects also received additional information about the percentage of their correct responses (CRs) and the reaction time (RT) average of their responses. In the test phase, participants performed eight blocks of the 2 and 3-back task. Subjects had no feedback during this time. Their results on this test were useful to evaluate their progress on n-back, and they are reported below and in Supplementary Table 1. The nontrained group did nothing during the training period. See also supplementary material and Supplementary Figure 1 for a more specific description of the n-back training protocol.

### 2.5. Neuroimaging Analysis

Preprocessing and statistical analysis of fMRI data were conducted with SPM12 (Wellcome Trust Centre for Neuroimaging, London, UK). The personnel responsible for fMRI data analysis were also blinded to the participants' group assignment. Preprocessing included the following steps: head motion correction, where the functional images were realigned and resliced to fit the mean functional image. No participant had a head motion of more than 1.5mm/degrees in any of the six directions during functional data recording. Afterwards, the anatomical image (T1-weighted) was coregistered to the mean functional image, and the transformed anatomical image was then segmented. The functional images were spatially normalized to the MNI (Montreal Neurological Institute, Montreal, Canada) space with a 3 mm3 resolution and spatially smoothed with an isotropic Gaussian kernel of 8 mm FWHM (Full-Width at Half-Maximum).

A general lineal model was used in the first level of analysis to obtain the task activation maps for each WM condition, compared to the control condition: “2-back>0-back”, “3-back>0-back”. The BOLD signal was estimated by convolving the stimuli onset with the canonical hemodynamic response function. Six motion realignment parameters were included as covariates of no interest in order to explain signal variations due to head motion. A high-pass filter (128s) was applied to the functional data to eliminate low-frequency components.

In a second level of analysis, whole-brain one-sample t-tests were conducted to study the brain regions involved in each condition (2-back>0-back and 3-back>0-back) for each group, using the fMRI data collected in S1. S1 data were also used to perform a Full Factorial ANCOVA (sex and age as a covariates) to examine the initial equality of the brain responses between the groups and the assigned experimental condition (Group x Training). Additionally, we used a Flexible Factorial design, with sex and age as covariates (repeated-measures ANCOVA), to study immediate effects of the training on the brain, comparing S2 to S1. An interaction analysis (Group x Training x Session) was carried out separately for each experimental condition (2-back and 3-back). Due to the data loss in S3, a separate Flexible Factorial analysis was performed to study the stability of the training effects 35 days after the training session (S1 vs S3). Results were p<0.05 FWE-cluster corrected with an auxiliary threshold of p<0.001. Finally, partial correlations with sex and age as covariates were performed to investigate the possible relationship between participants' performance (the difference obtained between S1 vs S2 in mean correct responses (CRs) and mean reaction times (RTs)) and the mean significant BOLD signal, i.e., the corresponding Eigen values for the significant clusters obtained in the prior Flexible Factorial analyses for each condition (2 and 3-back).

In all patients, T1-hypointense lesions were identified and filled using the “LST: Lesion Segmentation Tool” (https://www.applied-statistics.de/lst.html), calculating the lesion probability maps with the Lesion growth algorithm (LGA) [27]. The brain parenchymal fraction (BPF) for all the participants was obtained from the 3D image by following the SPM12 software (Wellcome Trust Centre for Neuroimaging, London, UK) segmentation step, according to the procedure described by Sanfilipo et al. [28].

### 2.6. Behavioral Analysis

SPSS 22 (IBM Corp) was used to process the neuropsychological and clinical data presented in Table 1 and the fMRI data obtained in each group in each session. 2x2 ANCOVAs [Group (HC vs MS) x Training (untrained vs trained); covariates: sex and age] were conducted to assess potential baseline differences among groups and training conditions. Additionally, 2x2x3 mixed model ANCOVAs [Group (HC vs MS) x Training (untrained vs trained) x Session (S1 vs. S2 vs. S3); covariates: sex and age] were conducted for each variable (CRs and RTs) and for each condition (2-back and 3-back). Longitudinal behavioral data loss was lower than that for neuroimaging data; thus, we lost 6 participants' data in all (2 participants in the HCt group, and 2 in the MSu group, 2 in the MSu group; see also Figure 1). For this reason, we decided to use the mean CRs and RTs of each group in S3 to replace the S3 missing values. All the analyses were followed by Bonferroni post-hoc tests, which provide p-values adjusted for multiple comparisons.

Finally, with the test phase data from the training, repeated-measures ANCOVAs (age and sex as covariates) for each n-back condition (2-back and 3-back) and performance measures (CRs and RTs for correct responses) were conducted separately, with group (HCt and MSt) as between-subject factor and tests from the training sessions (T1 vs T2 vs T3 vs T4) as within-subjects factor. To evaluate the improvement due to the task training, we carried out paired t-tests, comparing Test 1 with Test 4.

## 3. Results

### 3.1. Demographic and Neuropsychological Results

As Tables 1(a) and 1(b) show, no statistically significant differences between groups (HCu, HCt, MSu, and MSt) were observed for age, gender, cognitive performance, or fatigue scores. Conversely, statistically significant differences between groups were observed for BDI scores and, as expected, BPF volume.

### 3.2. Imaging Data Results

**Figure 2:**
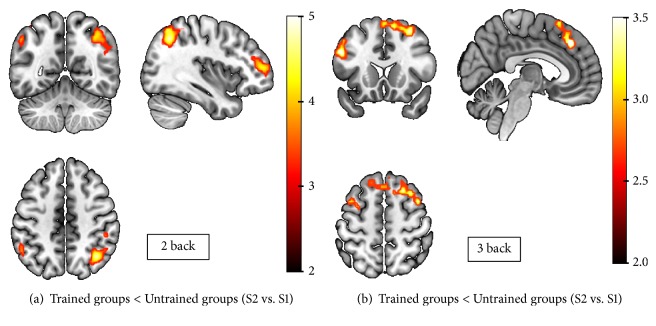
Neuroimaging results. (a) depicts the decreased activation (S2<S1) in frontoparietal areas observed during 2-back performance in trained groups as compared to untrained groups (FWEc=75, p<0.001). (b) shows the decreased frontoparietal activations (S2<S1) of trained groups as compared to untrained groups during 3back performance (FWEc=183, p<0.005).

As Supplementary Figure 1 reveals, during the performance of the 2 and 3-back tasks in the baseline session (S1), all participants showed brain activations in frontoparietal areas related to WM processes, and no differences between groups were observed. As Figure 2(a) and Supplementary Figure 2A show, when the activations associated with the performance of the 2-back task in S2 were compared to those observed in S1, both (HC and MS) trained groups showed reduced activation in the frontoparietal network, compared to the untrained groups.

As specified in Table 2, during the 2-back task, trained participants showed reduced activation in the left postcentral gyrus, right frontal gyrus, angular and supramarginal gyrus, and bilateral inferior parietal lobule. Similar decreased activation in trained groups was observed during the performance of the 3-back task (Figure 2(b), Supplementary Figure 2B), but in this case, these decreased activations were restricted to bilateral frontal areas, and they were especially remarkable in bilateral supplementary motor areas and bilateral middle and superior frontal gyrus (See Table 2). fMRI results comparing S3 to S1 are reported in the supplementary material (see Supplementary Table 2 and Supplementary Figure 3).

### 3.3. N-Back fMRI Task Performance

**Figure 3:**
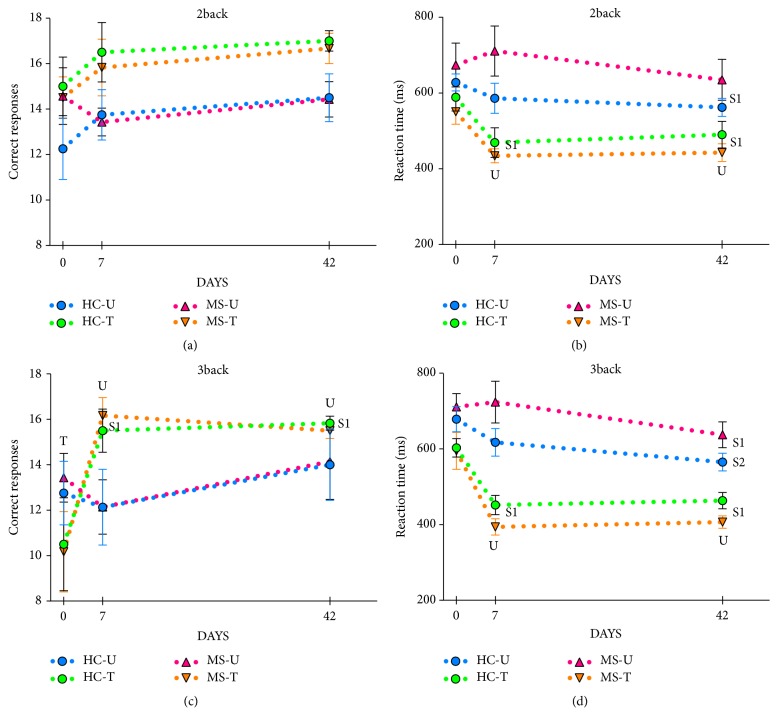
Behavioral results. Panels depict mean ± SEM of correct responses (CRs) and reaction times (RTs) for correct responses in the 2-back and 3-back tasks at each session. Trained groups exhibited shorter RTs but not an increased number of CRs (a) in the 2-back task at the posttraining (S2) and in the follow-up (S3) sessions than in the baseline (S1) session. Trained groups exhibited higher number of CRs (c) and shorter RTs (d) in the 3-back task at S2 and S3 than in S1 (U: different from untrained groups; T: different from trained groups; S1: different from baseline; S2: different from posttraining session; see Tables 3 and 4 for details).

As Table 3(a) and Figure 3 show, only the training factor yielded a statistically significant effect on the number of CR on the 2-back task. Conversely, the RT on the 2-back task was affected by the training effects and session factors as well as their interaction (Table 3(b)). Post-hoc comparisons revealed a RT for correct responses decrease in trained (but not in untrained) groups across sessions. Thus, although they had similar RTs in the baseline session, trained groups showed a statistically significant faster performance on the 2-back task than untrained groups in S2 and S3.

Both the number of CRs and the RTs on the 3-back task (Tables 4(a) and 4(b), Figure 3) were mainly dependent on a session x training condition interaction. Thus, only trained groups exhibited statistically significant increases in CR and accompanying reductions in RTs for correct responses across sessions. Consequently, although trained groups had similar RTs and a slightly lower number of CRs in the baseline session than the untrained groups, trained groups exhibited shorter RTs and a larger number of CRs in S2 and S3.

### 3.4. N Back Training Performance Test

The 2-back repeated measures ANOVA revealed a significant effect on CRs of training sessions (F_3,81_=3.86, p<0.05, and *ɳ*^2^=0.114), but not group (F_1,27_=0.046, p=0.831, and *ɳ*^2^=0.002) or the first-order interaction (F_3,81_=1.32, p=0.273, and *ɳ*^2^=0.047). Paired t-tests showed an increased number of CRs in T4 than in T1 (t=2.479, p<0.05).

In the same way, the 2-back repeated measures ANOVA revealed a significant effect on RTs for correct responses of training sessions (F_3,81_=10.72, p<0.001, and *ɳ*^2^=0.284), but not group (F_1,27_=0.022, p=0.883, and *ɳ*^2^=0.001) or the first-order interaction (F_3,81_=1.04, p=0.378, and *ɳ*^2^=0.037). Paired t-tests showed shorter RTs in T4 than in T1 (t=-4.33, p<0.001).

The 3-back repeated measures ANOVA revealed a significant effect on CRs of training sessions (F_3,81_=10.05, p<0.001, and *ɳ*^2^=0.271), but not group (F_1,27_=0.087, p=0.770, and *ɳ*^2^=0.003) or the first-order interaction (F_3,81_=0.746, p=0.528, and *ɳ*^2^=0.027). Paired t-tests showed an increased number of CRs in T4 than in T1 (t=3.89, p<0.001).

Similarly, the 3-back repeated measures ANOVA revealed a significant effect on RTs for correct responses of training sessions (F_3,81_=7.15, p<0.001, and *ɳ*^2^=0.209), but not group (F_1,27_=0.270, p=0.608, and *ɳ*^2^=0.010) or the first-order interaction (F_3,81_=1.04, p=0.377, and *ɳ*^2^=0.037). Paired t-tests showed shorter RTs in T4 than in T1 (t= -3.48, p<0.01).

### 3.5. Relationship between the Effects of Training on Cerebral Activation and Task Performance

The S2 vs S1 decrease in activation in the right medial frontal gyrus during 2-back performance was significantly correlated with the corresponding RT decreases for correct responses between these sessions (r=0.346, p<0.01). Moreover, the S2 vs S1 decrease in activation focused on the right superior frontal gyrus correlated with the RT for correct responses reduction during the performance of the 3-back task in these sessions (r=0.372, p<0.01). Finally, despite a significant reduction in the number of data due to technical problems (see methods section), a large and direct correlation (r=0.492, p<0.05) between S3 vs S1 decrease in activation in the right superior frontal gyrus was observed during 2-back performance.

## 4. Discussion

The main outcome of this randomized controlled study was the confirmation of the efficacy of a WM training program in HC and MS patients. More specifically, after four days (60 min/ day) of n-back training, both MS patients and HC showed a significant improvement in their performance on the WM trained task. Moreover, these performance improvements were accompanied by a significant decrease in brain activity in some frontoparietal areas belonging to the WM network. These results suggest that this training program increases n-back task performance and neural efficiency.

Thus, compared to the nontrained groups of MS patients and HC, trained (HC and MS patients) participants exhibited lower S2 RTs and an increase in CRs during the performance of the 2- and 3-back tasks. In addition, during the performance of the 2-back task, trained participants showed decreased activation in bilateral frontoparietal areas, which belong to the previously described WM network [20, 29]. A similar reduction in brain activity, although in this case restricted to bilateral frontal areas of the same network, was observed during the performance of the 3-back task. Interestingly, the beneficial effects of this training program were persistent over time. Thus, more than 42 days after the end of S3, WM performance in trained participants remained as good as immediately after completing the training program (S2), and it was significantly higher than before its implementation (S1). In S3, enhanced performance was again accompanied by a significant brain activity reduction in right frontoparietal areas. These findings are consistent with those of a similar study that reported improved performance and decreased activation in frontal and parietal areas after n-back training in young healthy volunteers [20] and they might be interpreted in the context of the “neural efficiency hypothesis” [20, 30].

The concept of neural efficiency refers to achieving maximum performance levels while deploying a minimum amount of brain resources, and it has received experimental support from previous studies involving healthy volunteers [20, 30, 31] and MS patients [32]. The training program used in the present study increased both task performance and neural efficiency. Direct support for this conclusion is provided by the observed statistically significant correlations between the reduction in the activation in frontal areas and the reduction in the RTs during the performance of 2 and 3-back tasks. In particular, we observed that the S2 vs S1 reduction in right medial frontal gyrus activity is strongly related to the S2 vs S1 decline in RTs on the 2-back tasks. A similar correlation was observed between S2 vs S1 activity reduction in the right superior frontal gyrus and in the RTs observed during the performance of the 3-back task. Moreover, despite a significant reduction in the number of data due to technical problems (see methods section), a large and direct correlation (0.492, p<0.05) between S3 vs S1 decrease in activation in the right precentral gyrus and the S3 vs. S1 reduction in the 3-back RTs was observed. Taken together, these results confirm that the training program used in the present study enhances both performance on the n-back task and neural efficiency.

However, it should be noted that our results and their interpretation seem to be at odds with those of other previous studies exploring neuroplasticity changes in response to cognitive rehabilitation in MS patients [8, 15, 33–36]. These studies have generally observed an improvement in cognitive performance, but at the cost of a higher degree of brain activation, that is, the exact opposite of what we observed in the present study. This discrepancy seems to be due to the distinct clinical status of the MS patients in each case. Indeed, when this variable and the inverted-U relationship between brain activation and cognitive performance observed throughout MS progression [37, 38] are taken into account, all these results seem to conform a coherent picture. Thus, it should be noted that we recruited a homogeneous group of MS patients with the same clinical phenotype (RR) and no cognitive impairment and, even more importantly, we chose MS patients who did not achieve this normal cognitive performance through the early spontaneous adaptive neuroplasticity processes (e.g., increased brain activation) described in previous studies [4, 32, 39]. Therefore, the patients recruited in our study were very similar to HC, and the expected effects of n-back training should also be the same as in HC, namely, an increase in performance and neural efficiency [20, 32, 38]. Conversely, the previous studies referred to above recruited patients with more advanced MS who exhibited moderate cognitive impairment. In these mildly impaired MS patients, increased cognitive performance is expected to be achieved only if additional brain resources can be recruited [32, 38, 40], which is exactly what was observed after they received neuropsychological training [8, 15, 33, 34, 36, 41].

On the other hand, it should also be noted that our study differs from others [8, 15, 33–36] in another aspect. Whereas our training program was designed to improve a specific cognitive domain (WM), previous studies tried to improve several cognitive functions at the same time. However, the possible relevance of this procedural difference, if any, when trying to explain the distinct dynamics of brain activation after cognitive training, remains unknown. By contrast, it might safely be concluded that repeated practice of the n-back task, which involves information maintenance and manipulation and also a substantial information processing speed demand, results in improved n-back performance, enhanced information processing speed (as revealed by RT reduction for correct responses), and consistent cerebral changes in the WM frontoparietal network.

Finally, the present study also presents some limitations that should be pointed out. First, as in most of the preceding studies exploring the beneficial effects of cognitive training in MS patients [18, 42], the present results were obtained in a small sample of cognitively preserved participants, hence reducing the statistical power of our study and the generalizability of its results. The small sample problem was further aggravated by the loss of a significant proportion of participants' data from S3. Second, as discussed above, the results of the present study were obtained with a carefully selected and homogeneous group of MS patients without cognitive impairment. Third, in this study we used repeated n-back training that is not directly comparable to previous studies using other rehabilitation programs aimed to improve a wide range of cognitive functions. Therefore, these results should not be overgeneralized, and further studies are needed to determine whether this specific training program is also useful for improving other cognitive skills (performance transfer effects) in MS patients with cognitive impairment and apparent brain damage [18, 30]. Fourth, we did not assess this possible generalization effect to other tasks, cognitive functions, or daily life activities, which are important issues that should be explored in future studies with a large sample of MS patients. Finally, this clinical trial was not preregistered in a public database, a practice increasingly recommended to increase research transparency.

## 5. Conclusions

In summary, the results of this study show that n-back training enhances performance and decreases the cerebral activation of WM networks in cognitively preserved MS patients and HC patients. These findings are promising and warrant further studies assessing the effects of this training in larger cohorts of MS patients with different degrees of cognitive impairment.

## Figures and Tables

**Table:** (a) Demographic and clinical data of all participants.

Results	HC_U_ (n=15) (mean± SD)	HC_T_ (n=14) (mean± SD)	MS_U_ (n=14) (mean± SD)	MS_T_ (n=15) (mean± SD)	Differences
Age	34.13±6.07 (25-45)	31.21±8.72 (24-50)	36.14±5.97 (22-46)	35.80±7.3 (22-46)	Training: F_1,54_= 0.762, p= 0.387 Group: F_1,54_= 3.115, p=0.083 Interaction: F_1,54_= 0.475, p=0.494

Gender (men/women)	9/6	6/8	3/11	7/8	*χ* ^2^_(3)_=4.51, p=0.212

Educational level (1-6 levels)	3.73±1.28	4.71±0.83	3.71±1.49	3.73±1.71	Training: F_1,54_= 1.925, p= 0.171 Group: F_1,54_= 1.925, p=0171 Interaction: F_1,54_= 1.781, p=0.188

EDSS	-	-	1.80±1.70	1.67±1.51	t_27_=0.19, p = 0.848
Mean years disease duration	-	-	7.54±5.12	8.33±5.96	t_27_= -0.38, p = 0.850
Total lesion volume (mL)	-	-	4.39±4.88	2.36±3.56	t_27_ = 1.28, p= 0.210

BPF	0.86±0.01^C,D^	0.85±0.02^C,D^	0.84±0.02	0.84±0.01	Training: F_1,54_= 0.188, p= 0.666 *Group: F*_*1,54*_*= 10.301, p=0.002* Interaction: F_1,54_= 0.367, p=0.547

BDI	7.85±5.65^C,D^	4.50±5.24^C,D^	14.21±7.98	11.47±8.33	Training: F_1,54_= 1.844, p= 0.181 *Group: F*_*1,54*_*= 8.831, p=0.005* Interaction: F_1,54_= 0.018, p=0.894

FSS	-	-	47.36±16.01	40.80±17.98	t_27_=1.03, p=0.310

HCu: HC untrained group; HCt: HC trained group; MSu: MS untrained group; MSt: MS trained group; Educational level: 1= Primary education, 2=Lower secondary education, 3=Upper secondary education, 4=Post-secondary education non-tertiary, 5=First stage of tertiary education, 6=Second stage of tertiary education; EDSS: Expanded Disability Status Scale; BPF: Brain Parenchymal Fraction; BDI: Beck Depression Inventory; FSS: Fatigue Severity Scale.  ^A^denotes statistically significant different from the HCu group;  ^B^denotes statistically significant different from the HCt group;  ^C^denotes statistically significant different from the MSu group;  ^D^denotes statistically significant different from the MSt group.

**Table:** (b) Neuropsychological performance data of all participants at baseline

Results	HC_U_ (n=15) (mean± SD)	HC_T_ (n=14) (mean± SD)	MS_U_ (n=14) (mean± SD)	MS_T_ (n=15) (mean± SD)	Differences
*BRN-B*					
PASAT (%)	76.22±8.87	78.33±17.45	74.86±18.18	83.44±13.01	Training: F_1,54_= 1.398, p= 0.244 Group: F_1,54_= 0.172, p=0.681 Interaction: F_1,54_= 0.511, p=0.479
SDMT	59.69±9.09	66.17±6.37	54.93±10.56	60.80±10.13	Training: F_1,54_= 3.813, p= 0.056 Group: F_1,54_= 3.115, p=0.083 Interaction: F_1,54_= 0.475, p=0.494
SRT Long-Term Storage	58.46±8.08	52.67±12.94	52.07±13.53	52.53±10.29	Training: F_1,54_= 0.598, p= 0.444 Group: F_1,54_= 0.895, p=0.349 Interaction: F_1,54_= 0.823, p=0.369
SRT Consistent Long-Term Retrieval	51.31±11.76	47.83±5.71	43.21 ±14.75	42.07±12.32	Training: F_1,54_= 0.314, p= 0.578 Group: F_1,54_= 2.822, p=0.100 Interaction: F_1,54_= 0.080, p=0.779
SRT Delayed Recall	10.23±1.92	10.50±1.98	9.50 ±2.85	10.27±1.98	Training: F_1,54_= 0.552, p= 0.462 Group: F_1,54_= 0.478, p=0.493 Interaction: F_1,54_= 0.127, p=0.723
SPART Long-Term Storage	20.62±6.64	23.33±3.26	20.57±5.06	20.33±5.18	Training: F_1,54_= 0.549, p= 0.463 Group: F_1,54_= 0.827, p=0.368 Interaction: F_1,54_= 0.780, p=0.382
SPART Delayed-Recall	7.08±2.72	8.67±1.75	7.07±2.12	7.27±1.83	Training: F_1,54_= 1.748, p= 0.193 Group: F_1,54_= 1.084, p=0.304 Interaction: F_1,54_= 1.067, p=0.307
WLGT	22.54±3.57	25.17±3.66	21.14±6.29	21.40±5.58	Training: F_1,54_= 0.821, p= 0.370 Group: F_1,54_= 2.627, p=0.112 Interaction: F_1,54_= 0.554, p=0.461
*Matrix Subtest (WAIS III)*	105.71±14.79	106.43±16.34	111.15 ± 7.95	106.33±12.17	Training: F_1,54_= 0.335, p= 0.565 Group: F_1,54_= 0.567, p=0.455 Interaction: F_1,54_= 0.608, p=0.439

HCu: untrained group; HCt: HC trained group; MSu: MS untrained group; MSt: MS trained group. BRN-B: the Brief Repeatable Battery of Neuropsychological Test; SDMT: Symbol Digit Modalities Test; PASAT: Paced Auditory Serial Addition Test; SRT: Selective Reminding Test; SPART: Spatial Recall Test; WLGT: Word List Generation Test.

**Table 2:** List of brain activations as a result of the posttraining session (S2) in 2-back and 3-back load level between groups comparisons. Results were p<0.05 FWEc cluster-corrected using a threshold of p <0.001 at the uncorrected level and a cluster extension of k= voxels, respectively. (MNI: Montreal Neurological Institute coordinates; S1: basal session; S2: follow-up session 2.

Anatomical regions	K_voxels_	MNI coordinates			T
		*x*	*y*	*z*	
*2back: Trained groups < Untrained groups (S2vs.S1)*					
R Angular gyrus	319	36	-61	44	5.01
R Supramarginal gyrus		*48*	*-37*	*41*	*3.99*
R Inferior parietal lobule		*48*	*-43*	*56*	*3.93*
R Middle frontal gyrus	130	39	53	8	4.53
L Inferior parietal lobule	75	-54	-55	41	4.04
L Postcentral gyrus		*-45*	*-37*	*62*	*3.53*
*3back: Trained groups < Untrained groups (S2vs.S1)*					
R Superior medial frontal gyrus	320	6	35	41	4.15
R Middle frontal gyrus		*42*	*8*	*59*	*3.77*
R Superior frontal gyrus		*24*	*23*	*53*	*3.58*
L Superior frontal gyrus		*-15*	*29*	*53*	*3.57*
R Supplementary motor area		*9*	*20*	*62*	*3.44*
L Supplementary motor area		*0*	*17*	*62*	*3.25*
L Inferior frontal	183	-51	17	29	3.82
L Precentral gyrus		*-48*	*11*	*47*	*3.78*
L Middle frontal gyrus		*-42*	*8*	*56*	*3.64*
L Inferior frontal gyrus		*-45*	*14*	*14*	*2.88*

**Table:** (a) 2-back correct responses. Omnibuses of the ANCOVA (covariates: sex and age) for the number of correct responses during 2-back performance. Descriptive statistics are provided as mean and standard deviation. Statistically significant effects are highlighted in bold.

2-BACK CORRECT RESPONSES				
MAIN EFFECTS	DESCRIPTIVE STATISTICS			
Group (F_1,52_= 0.686, p=0.41, *η*^2^ =.013)				
**Training (F**_**1,52**_ **= 7.12, p<0.01, ** **η** ^2^ **=0.12)**	*UNTRAINED*	*S1*	*S2*	*S3*
Session (F_2,51_ =2.85, p=0.07, *η*^2^ =0.10)	*Mean*	14.07	14.76	14.89
INTERACTIONS	*SD*	3.53	2.46	3.13
Training x Session				
(F_2,51_ =1.88, p=0.16, *η*^2^ =0.07)				
Training x Group	*TRAINED*	*S1*	*S2*	*S3*
(F_2,51_ =0.017, p=0.90, *η*^2^ =0.00)	*Mean*	14.41	16.62	16.80
Session x Group	*SD*	2.34	1.99	1.13
(F_2,51_ =0.001, p=0.99, *η*^2^ =0.00)				
Training x Session x Group				
(F_2,51_ =0.126, p=0.88, *η*2 =0.005)				

**Table:** (b) 2-back reaction times for correct responses. Omnibuses of the ANCOVAs (covariates: sex and age) for the reaction times during 2-back performance. Within and between comparisons were conducted using Bonferroni post-hoc tests, and their corresponding significance levels (p) and effect sizes (Cohen's d and its 95% confidence interval) are reported. Statistically significant effects are highlighted in bold. (U, untrained; T, trained).

2-BACK REACTION TIMES FOR CORRECT RESPONSES								
MAIN EFFECTS	DESCRIPTIVE STATISTICS AND MEANS COMPARISONS							
Group (F_1,52_= 1.72, p=0.19, *η*^2^ =0.03)	*UNTRAINED*	*S1*	*S2*	*S3*	WITHIN GROUPS - UNTRAINED			
**Training (F**_**1,52**_ **= 15.70, p<0.001, ** **η** ^2^ ** =0.23)**	*Mean*	662.38	636.48	605.37	*S1-S2*	p=0.31	d= 0.20	(-0.53, 0.93)
**Session (F**_**2,51**_ ** = 7.27, p<0.01, ** **η** ^2^ ** =0.22)**	*SD*	123.40	140.17	147.74	***S1-S3***	**p<0.05**	**d= 0.42**	**(-0.32, 1.16)**
INTERACTIONS					*S2-S3*	p=0.31	d= 0.22	(-0.51, 0.95)
**Training x Session**	*TRAINED*	*S1*	*S2*	*S3*	WITHIN GROUPS -TRAINED			
**(F**_**2,51**_ ** = 10.18, p<0.001, ** **η** ^2^ ** =0.29)**	*Mean*	615.99	475.45	492.48	***S1-S2***	**p<0.001**	**d= 1.23**	**(0.43, 2.02)**
Training x Group	*SD*	134.11	91.03	92.63	***S1-S3***	**p<0.001**	**d= 1.07**	**(0.29, 1.85)**
(F_2,51_ =1.93, p=0.17, *η*^2^ =0.04)					*S2-S3*	p=1	d= -0.18	(-0.91, 0.54)
Session x Group	BETWEEN GROUPS ( U vs. T)							
(F_2,51_ =1.23, p=0.30, *η*^2^ =0.05)	*S1*		*S2*	*S3*				
Training x Session x Group	p=0.14		**p<0.001**	**p<0.001**				
(F_2,51_ =0.37, p=0.69, *η*^2^ =0.014)	d= 0.36		**d= 1.36**	**d=0.92**				
	(-0.37, 1.09)		**(0.55, 2.17)**	**(0.15, 1.68)**				

**Table:** (a) 3-back correct responses. Omnibuses of the ANCOVA (covariates: sex and age) for the number of correct responses during 3-back performance. Within and between comparisons were conducted using Bonferroni post-hoc tests, and their corresponding significance levels (p) and effect sizes (Cohen's d and its 95% confidence interval) are reported. Statistically significant effects are highlighted in bold (U: untrained; T: trained).

3-BACK CORRECT RESPONSES								
MAIN EFFECTS	DESCRIPTIVE STATISTICS AND MEANS COMPARISONS							
Group (F_1,52_= 0.72, p= 0.40, *η*^2^ = 0.014)	*UNTRAINED*	*S1*	*S2*	*S3*	WITHIN GROUPS - UNTRAINED			
Training (F_1,52_= 2.78, p= 0.10, *η*^2^= 0.51)	*Mean*	13.14	12.93	14.06	*S1-S2*	p=1.00	d= 0.06	(-0.67, 0.79)
Session (F_2,51_= 1.18, p= 0.31, *η*^2^ = 0.04)	*SD*	3.62	3.52	4.12	*S1-S3*	p=0.72	d= -0.24	(-0.97, 0.49)
INTERACTIONS					*S2-S3*	p=0.072	d= -0.29	(-1.03, 0.44)
**Training x Session**	*TRAINED*	*S1*	*S2*	*S3*	WITHIN GROUPS - TRAINED			
**(F**_**2,51**_ ** =19.81, p<0.001, ** **η** ^2^ ** =0.44)**	*Mean*	11.10	16.41	16.47	***S1-S2***	**p<0.001**	**d= -1.71**	**(-2.57, 0.86)**
Training x Group	*SD*	4.08	1.59	1.56	***S1-S3***	**p<0.001**	**d= -1.74**	**(-2.59, 0.88)**
(F_1,52_ =0.22, p=0.64, *η*^2^ =0.004)					*S2-S3*	p=1.00	d= -0.04	(-0.77, 0.69)
Session x Group	BETWEEN GROUPS ( U vs. T)							
(F_2,51_ =0.20, p=0.82, *η*^2^ =0.008)	*S1*	*S2*		*S3*				
Training x Session x Group	**p=0.035**	**p<0.001**		**p<0.001**				
(F_2,51_ =1.62, p=0.21, *η*^2^ =0.06)	**d= 0.53**	**d= -1.27**		**d= -0.77**				
	**(-0.21, 1.27)**	**(-2.07, -0.48)**		**(-1.53, -0.02)**				

**Table:** (b) 3-back reaction times for correct responses. Omnibuses of the ANCOVA (covariates: sex and age) for the reaction times during 3-back performance. Within and between comparisons were conducted using Bonferroni post-hoc tests, and their corresponding significance levels (p) and effect sizes (Cohen's d and its 95% confidence interval) are reported. Statistically significant effects are highlighted in bold. (U, untrained; T, trained).

3-BACK REACTION TIMES FOR CORRECT RESPONSES								
MAIN EFFECTS	DESCRIPTIVE STATISTICS AND MEANS COMPARISONS							
Group (F_1,52_ = 2.16, p=0.15, *η*^2^ =0.04)	*UNTRAINED*	*S1*	*S2*	*S3*	WITHIN GROUPS - UNTRAINED			
**Training (F**_**1,52**_ ** = 22.39, p<0.001, ** **η** ^2^ ** =0.30)**	*Mean*	705.36	664.29	610.16	*S1-S2*	p=0.126	d= -0.29	(-0.44, 1.02)
Session (F_2,51_ = 2.72, p=0.075, *η*^2^ =0.096)	*SD*	144.77	135.26	125.64	*S1-S3*	**p<0.001**	**d= -0.70**	**(-0.05, 1.45)**
INTERACTIONS					*S2-S3*	**p<0.01**	**d= -0.41**	**(-0.32, 1.15)**
**Training x Session**	*TRAINED*	*S1*	*S2*	*S3*	WITHIN GROUPS - TRAINED			
**(F**_**2,51**_ ** = 12.80, p<0.001, ** **η** ^2^ ** =0.33)**	*Mean*	634.93	461.81	479.10	***S1-S2***	**p<0.001**	**d= -1.31**	**(0.51, 2.11)**
Training x Group	*SD*	155.24	104.73	107.11	***S1-S3***	**p<0.001**	**d= -5.34**	**(3.79, 6.90)**
(F_1,52_ =0.69, p=0.41, *η*^2^ =0.01)					*S2-S3*	p=1.00	d= -1.17	(0.38, 1.96)
Session x Group	BETWEEN GROUPS ( U vs. T)							
(F_2,51_ =0.81, p=0.45, *η*^2^ =0.03)	*S1*	*S2*		*S3*				
Training x Session x Group	p=0.071	**p<0.001**		**p<0.001**				
(F_2,51_ =0.11, p=0.89, *η*^2^ =0.005)	d= 0.47	**d= 1.67**		**d= 1.12**				
	(-0.27, 1.21)	**(0.83, 2.52)**		**(0.34, 1.91)**				

## Data Availability

The behavioral and imaging data corresponding to the findings of the present study are available from the corresponding author upon request.

## Supplementary Materials

Supplementary Materials Supplementary Table 1. Mean and standard deviations (mean±SD) of performance in tests of each training session (TS). Supplementary Table 2: List of brain activations as a result of the posttraining session (S3) in 2-back load level between groups comparisons. Supplementary Figure 1. N-back training protocol. Supplementary Figure 2. Activations in frontoparietal areas for healthy controls (HC) and multiple sclerosis (MS) patients during WM performance. Supplementary Figure 3. Neuroimaging results as a result of the posttraining session (S3), compared to the basal session (S1), in 2-back load level between-group comparisons. Supplementary Figure 4. Activity changes in frontoparietal areas during WM performance.
