# Supplementary material for: Repeated Working Memory Training Improves Task Performance and Neural Efficiency in Multiple Sclerosis Patients and Healthy Controls
Source: Mult Scler Int. 2019 Apr 16;2019:2657902. doi: 10.1155/2019/2657902 (PMC6500632; doi:10.1155/2019/2657902)

N-back training protocol

The training group carried out four consecutive sessions of *n*-back training after fMRI Session 1, on laptops placed in our laboratory at the University.

Participants performed one training session per day. Each training session lasted 60 minutes and was distributed in two phases: the learning part and the testing part (see supplementary Figure 1). During the learning part, participants performed an adaptative n-back paradigm adapted from Jaeggi et al. (2008) for about 60 minutes (3 n-back runs of 15 minutes), whereas in the testing part, participants performed a simple n-back task that lasted 10 minutes. During the learning part, three working memory loads were presented, 1, 2, and 3 back. Participants pressed the “yes” button when the current letter shown on the screen matched the one presented 1, 2, or 3 items back, and they pressed “no” when there were no targets. As on the n-back fMRI task, participants were instructed to give manual responses only with their right hand, responding to targets with their thumb and to non-targets with their forefinger. Feedback was introduced after each response: a coloured circle appeared for a few seconds at the corner of the screen: green if the answer was correct, red if it was an error, and blue if participants did not press any button. Furthermore, at the end of each block, subjects received information about their performance: correct response percentage and reaction time average.

During the learning part, we changed the level of difficulty by changing the level of “n” (1, 2, or 3) in order to motivate participants to improve. After each block, the participant’s individual performance was analysed, and the *n*-back level was automatically adjusted. Thus, if the participant had at least 90% correct answers, the level of “n” in the next block was increased by one, but it was decreased by one if accuracy was below 80%. In all other cases, the *n*-level remained constant. In the last run, we increased the percentage by five percent to make it more difficult. Each run started with the minimum level of “n” (1) for motivational reasons.

On test part 2, participants performed an eight-block *n*-back task, four for 2-back and four for the 3-back load level (the entire task lasted 10 minutes). Subjects had no feedback on this performance, and their results on this test were useful to evaluate their progress on *n*-back performance.

Supplementary Figure 1. N-back training protocol.

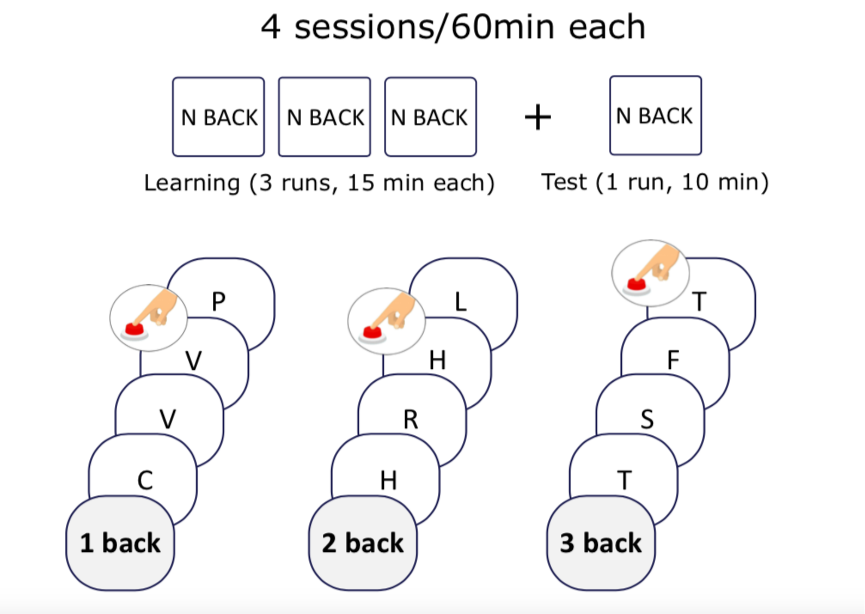

Supplementary Table 1. Means and standard deviations (mean±SD) of performance on tests in each training session (TS).

	2-back			
	CRs		RTs	
**TS**	**HCt**	**MSt**	**HCt**	**MSt**
**1**	21.07±2.81	20.27±3.82	501,53±124.46	483,11±77.00
**2**	21.79±3.16	21.40±2.56	469,12±90.58	472,97±97.99
**3**	22.29±1.68	21.80±2.18	436,61±67.17	459,62±20.93
**4**	21.57±2.28	22.60±1.60	423,97±79.16	433,35±74.92
	**3-back**			
**1**	18.07±4.23	18.80±3.99	492,31±147.15	500,22±109.81
**2**	19.43±4.36	20.40±3.42	468,27±85.53	458,18±91.56
**3**	21.64±1.65	20.93±3.33	422,59±103.07	457,12±88.72
**4**	21.29±1.44	21.40±1.72	411,90±96.45	448,62±90.68

TS=Training Session, CRs= Correct Responses, RTs=Reaction Times

Supplementary Figure 2. Activations in fronto-parietal areas for healthy controls (HC) and multiple sclerosis (MS) patients during WM performance. Panel A depicts the contrast 2-back> 0-back. Panel B depicts the contrast 3-back> 0-back. All results were $p<0.05$, FWEc corrected. Colour scale expresses the t-scores corresponding to these comparisons.

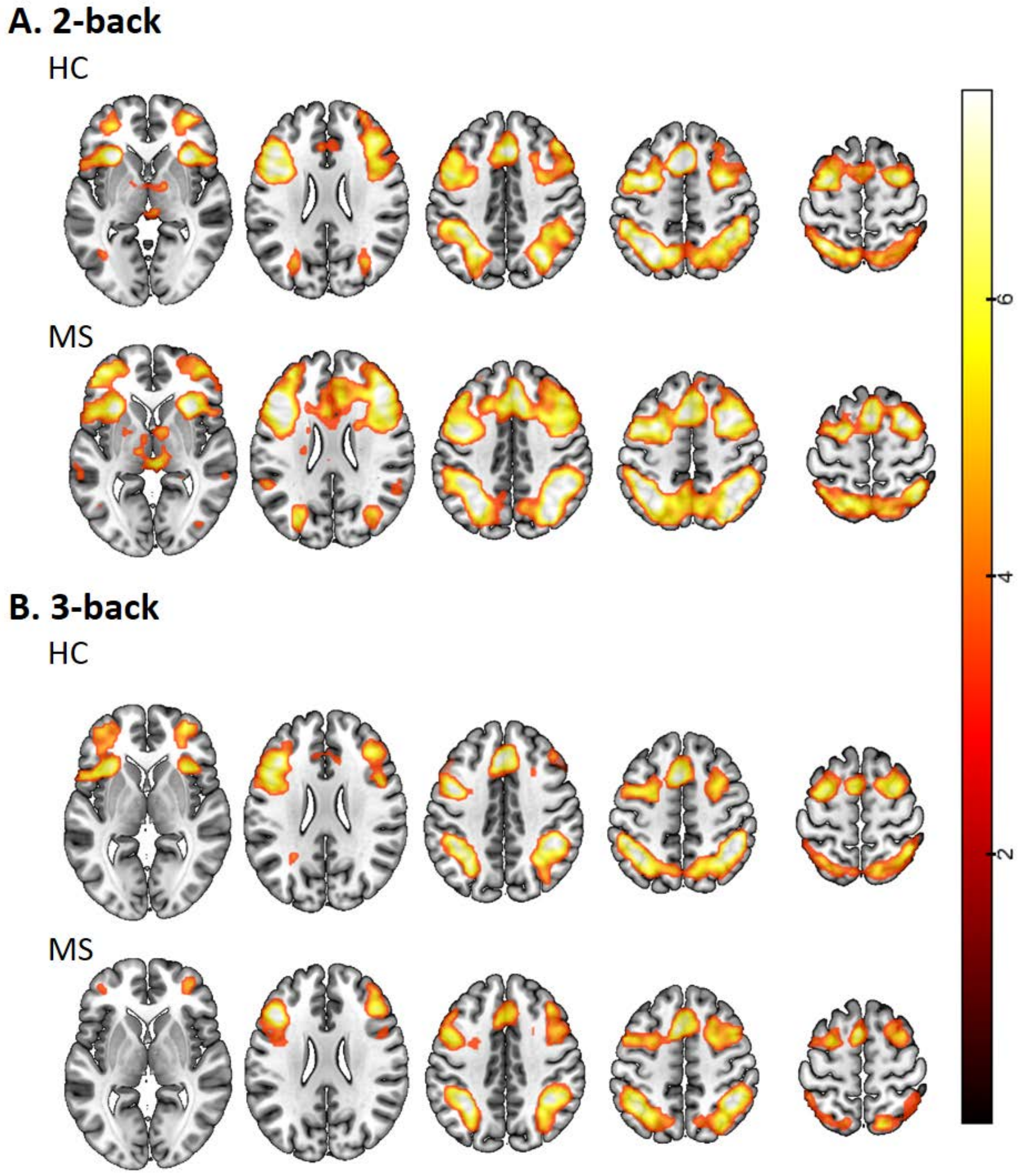

Supplementary Table 2: List of brain activations as a result of the post-training session (S3) in 2-back load level between-group comparisons. Results were p<0.05 FWEc cluster-corrected using a threshold of p <0.001 at the uncorrected level, and a cluster extension of k= voxels, respectively. (MNI: Montreal Neurological Institute coordinates; S1: basal session; S3: follow-up session 3. R: right, L: Left).

**Anatomical regions**	**K $_{voxels}$**	**MNI coordinates**			**T**
		x	*y*	*z*	
**2back: Trained groups < Untrained groups (S3vs.S1)**					
R Angular gyrus	157	39	-52	38	5.08
R Supramarginal gyrus		*60*	*-43*	*35*	*4.52*
R Inferior parietal lobule		*54*	*-40*	*47*	*4.30*
R Precentral gyrus	103	45	8	41	5.02
R Inferior frontal gyrus		*39*	*17*	*29*	*4.64*
R Middle frontal gyrus	91	33	53	23	4.49
R Middle cingulate gyrus	65	3	32	32	4.53
R Superior medial frontal gyrus		*9*	*23*	*44*	*3.91*

Supplementary Figure 3: Decreased activations (S3<S1) in frontoparietal regions of trained groups compared to untrained groups during 2-back performance (FWEc=65, p<0.001). Colour scale expresses the t-scores of these comparisons.

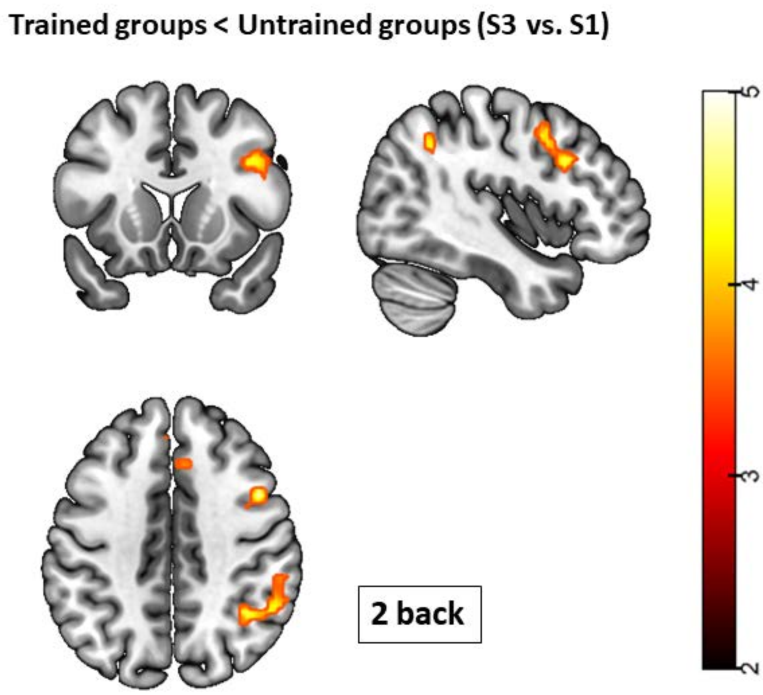

Supplementary Figure 4. Activity changes in fronto-parietal areas during WM performance. Panel A depicts the decrease of activity in fronto-parietal areas observed between the S2 and S1 sessions in trained (but not in untrained) groups during the performance of the 2-back task. Panel B shows similar activity decreases of fronto-parietal activity between S2 and S1 sessions in the trained (but not in the untrained) groups during 3-back task performance. Panel C illustrates the decreased activation observed in S3 as compared to S1 in trained (but not in untrained) groups during 2-back task performance. R: right; L: left.

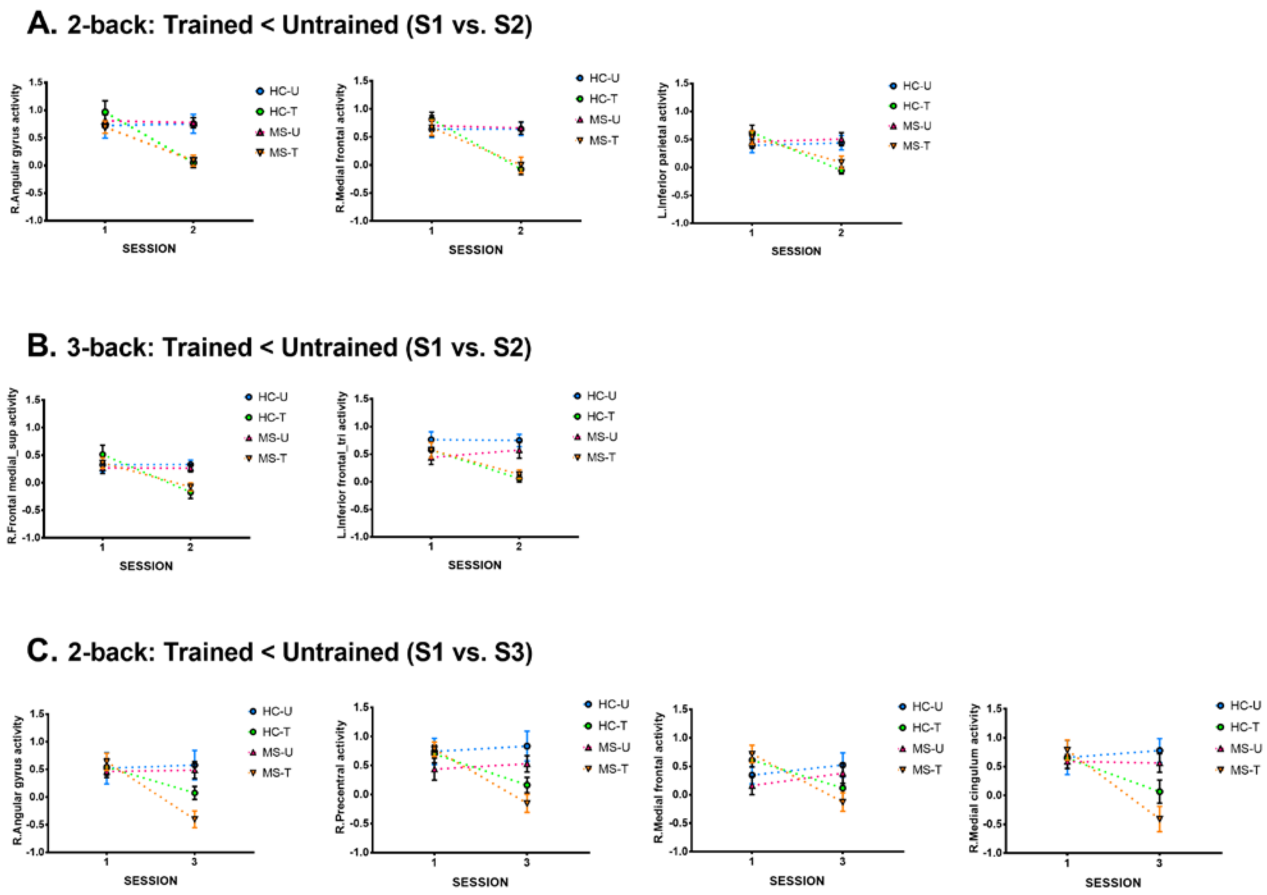

Supplement: Supplementary Materials — Supplementary Table 1. Mean and standard deviations (mean±SD) of performance in tests of each training session (TS). Supplementary Table 2: List of brain activations as a result of the posttraining session (S3) in 2-back load level between groups comparisons. Supplementary Figure 1. N-back training protocol. Supplementary Figure 2. Activations in frontoparietal areas for healthy controls (HC) and multiple sclerosis (MS) patients during WM performance. Supplementary Figure 3. Neuroimaging results as a result of the posttraining session (S3), compared to the basal session (S1), in 2-back load level between-group comparisons. Supplementary Figure 4. Activity changes in frontoparietal areas during WM performance. [file 2657902.f1.pdf]
